# Shedding new light on *Cyclospora*: how the use of ultraviolet fluorescence microscopy can improve diagnosis of cyclosporiasis

**DOI:** 10.1128/jcm.01084-24

**Published:** 2024-12-09

**Authors:** Angela Ma, Blaine A. Mathison, Marc Roger Couturier

**Affiliations:** 1 Department of Pathology, University of Utah 161530 , Salt Lake City, Utah, USA; 2 Institute for Clinical and Experimental Pathology, ARUP Laboratories 549001 , Salt Lake City, Utah, USA; Mayo Clinic Minnesota, Rochester, Minnesota, USA

**Keywords:** UV microscopy, coccidia, protozoa, *Cyclospora*, *Cystoisospora*

## Abstract

Cyclosporiasis has historically been underdiagnosed due to a lack of clinical recognition and challenges in laboratory detection. Microscopic detection of *Cyclospora* requires either the modified Kinyoun’s acid-fast or safranin stains, which are not part of the standard ova-and-parasite examination. Alternatively, the use of fluorescence microscopy for the detection of *Cyclospora* due to the organism’s autofluorescent properties has been an available tool in the field of clinical parasitology. However, it is unknown how ultraviolet (UV) fluorescence performs compared to permanent stains in routine diagnostic laboratory settings. In this study, we present a direct head-to-head comparison of UV fluorescence against modified acid-fast (MAF) stains using 50 (35 *Cyclospora* positive, 5 *Cystoisospora* positive, and 10 *Cystoisospora* negative) blinded, concentrated stool specimens. Five technically competent medical laboratory technologists of varying years of experience in clinical parasitology at a large reference laboratory independently read each of the MAF slides and UV wet mounts. Overall, the performance of UV fluorescence was similar to MAF slides with a higher agreement of 88.8% (95% CI 86.3%–91.3%) compared to 85.2% (95% CI 84.2%–86.2%), respectively. A higher false-negative rate was associated with MAF slides (MAF: 11.6%, UV: 6.8%). However, UV fluorescence was found to have a higher proportion of false positives (UF: 3.2%, MAF: 2%) and misidentified coccidia. Discrepant results regardless of methods were more frequently observed with technologists who have less experience in parasite morphology. This study is the first to document evidence to support the increased sensitivity and utility of UV fluorescence to improve laboratory diagnosis of cyclosporiasis.

**IMPORTANCE:**

This study is important as there is a dearth of studies in our field of clinical parasitology that investigate and establish performance characteristics of classic and newer, non-molecular methods. While these studies may not seem as heavy hitting as some new technologies described in our related disciplines, for our field, such studies are long overdue and critically lacking.

## INTRODUCTION

Human cyclosporiasis is caused by intestinal coccidian parasites in the genus *Cyclospora. Cyclospora cayetanensis* and *C. ashfordi* are widespread and responsible for most seasonal outbreaks in the United States while *C. henanensis* is known only from China (1). The parasites are broadly distributed worldwide and endemic to subtropical and tropic regions and in low-resource settings (2). Infection is typically attributed to ingestion of oocysts on contaminated produce or water. Public health and food safety attention to *Cyclospora* has risen drastically in the past few decades due to its association with seasonal foodborne outbreaks from contaminated fresh produce that are commonly consumed raw, such as berries, herbs, and leafy greens. The life cycle of *Cyclospora* involves sporulation of the oocyst in the environment for 2–4 weeks after passage from an infected host (3). Ingestion of a fully sporulated oocyst prompts sporozoites to excyst in the small intestine and cause intracellular infection of enterocytes in the duodenum. Asexual and sexual maturation of the parasite occurs within a vacuole, resulting in the production of either type I or type II meronts, respectively. Merozoites generated from asexual reproduction are responsible for perpetuating autoinfection in the host, and a fertilized zygote eventually matures into an unsporulated oocyst that is excreted into the environment to continue the life cycle in new hosts.

Patients infected with *Cyclospora* often present with watery diarrhea that may be accompanied by abdominal pain, nausea, fatigue, and fever (4, 5). Asymptomatic infection in immunocompetent patients from endemic regions has also been reported. Contrary to acute gastrointestinal disease caused by viral and bacterial pathogens, patients with cyclosporiasis may experience chronic or remitting cycles of diarrhea. Cyclosporiasis most commonly presents in returning travelers from areas where C*yclospora* is endemic or non-travelers with epidemiologic links to contaminated food or water. On rare occasions, extra-intestinal disease involving the biliary tract can occur and is more frequently observed in immunocompromised patients. Currently, there is no vaccine for cyclosporiasis. The primary form of treatment in immunocompetent patients is oral trimethoprim-sulfamethoxazole (4).

*Cyclospora* has been a long-standing under-diagnosed diarrheal pathogen due to several factors. Clinically, healthcare providers may not be aware that routine ova-and-parasite (O&P) examinations on stool specimens do not readily detect *Cyclospora* and other coccidian parasites. The stain of choice is the modified Kinyoun’s acid-fast or safranin, which may require additional ordering by the healthcare provider if *Cyclospora, Cystoisospora*, or *Cryptosporidium* infection is suspected (6, 7). Similarly, histopathological diagnosis of cyclosporiasis is not routine and can be difficult if pathologists lack the expertise to recognize *Cyclospora* in intestinal epithelial cells. The recent introduction of *Cyclospora* as a target in commercial multiplex nucleic acid amplification tests (NAATs) now allows for its increased diagnosis in laboratories that have adopted these tests to replace or supplement routine stool examinations (4).

Larger medical centers and reference laboratories may employ specialized permanent stains for *Cyclospora* and other coccidia. However, smaller laboratories may not have the capacity or skilled personnel to do so. Autofluorescence of *Cyclospora* oocysts is thus an attractive property to exploit for diagnostic purposes, providing a sensitive and simple method for laboratory detection. Direct examination by fluorescence microscopy of wet mounts prepared from stool specimens for *Cyclospora* is believed to be comparable to or more sensitive than permanent smears; however, no literature has evaluated the two diagnostic methods directly. In this study, we compare *Cyclospora* detection by ultraviolet (UV) fluorescence to modified acid-fast (MAF) permanent smears and emphasize the importance of morphological expertise in personnel working in clinical parasitology laboratories.

## MATERIALS AND METHODS

Stool specimens used in this study comprised random archived specimens submitted to the laboratory for analysis. Specimens were collected in various fixatives, including 10% formalin, TOTAL-FIX, EcoFix, and Alcorfix used for routine parasite detection that had been submitted for clinical parasitology testing in a national reference laboratory according to IRB #7275. In all, 50 blinded, concentrated specimens were included in this study to make wet mounts for UV microscopy, including 35 *Cyclospora*-positive specimens, 5 *Cystoisospora*-positive specimens, and 10 coccidia-negative specimens collected over a period of 3 years. Specimens were derived from multiple states. No mixed positive specimens were included in the study. Coccidia-positive specimens were enrolled from an archived collection of specimens previously tested and reported by the clinical parasitology laboratory. Positive specimens had previously been identified by either artificial intelligence-augmented trichrome stain with UV microscopy confirmation or MAF stain with UV microscopy. Stool specimens were centrifuged at 400 × *g* for 2 minutes using Parasep (Apacor, Wokingham, UK) tubes as per the manufacturer’s instructions for use. MAF slides for all 50 specimens were prepared as per standard staining procedures (https://www.cdc.gov/dpdx/diagnosticprocedures/stool/staining.html). All slides were air-dried and then coverslipped using a Tissue-Tek (Sakura Finetek, Torrance, CA) automated coverslipping instrument. Aliquots of concentrated stool specimens for UV wet mount preparation and corresponding MAF slides were randomly numbered from 1 to 100.

Five medical laboratory technologists technically competent in reading MAF and UV slides were chosen for participation in the study based on various levels of experience in the clinical parasitology laboratory; expertise ranged from highly experienced (technologist 1, > 5 years) to least experienced (technologist 5, < 6 months). Technologists were informed that they were participating in a stool UV evaluation study but did not know that specimens had paired MAF slides. Specimens for UV examination and MAF slides were provided to a single technologist for a 1-week period to independently read and record results for all specimens. Technologists were not permitted to share results. Technologists were instructed to prepare UV wet mount slides by placing 1 drop of the specimen with a disposable pipet onto a glass slide. Both the UV wet mount and MAF slides were evaluated as per standard clinical procedures. Technologists were instructed to document if a specimen was negative for coccidia, or if positive, to indicate whether *Cyclospora, Cystoisospora*, or both were detected. Technologists were not permitted to prepare a replicate UV wet mount or MAF slide if they were uncertain of coccidian positivity or asked another technologist for assistance.

The performance of UV wet mount and MAF slides when manually read by technologists was evaluated by determining the overall agreement between methods, and the number and proportion of false-negative, false-positive, and discrepant results, stratified by the technologist. All specimens for which none of the technologists accurately detected the correct coccidian or falsely reported as coccidia-negative were backread (blinded) by one of the authors with parasitology expertise.

## RESULTS

Across all technologists, UV wet mount preparations were slightly more accurate compared to MAF stained slides, with an overall agreement for UV and MAF methods at 88.8% (95% CI 86.3%–91.3%) and 85.2% (95% CI 84.2%–86.2%), respectively (Table 1). Higher rates of false negatives were observed for MAF slides (11.6%, 95% CI 11.1%–12.1%) compared to UV wet mount (6.8%, 95% CI 5.6%–8.0%) with the exception of technologist 5 (14% for UV, 10% for MAF). About 13 specimens were associated with false negatives (*n* = 11 for *Cyclospora* and *n* = 2 for *Cystoisospora*), of which 4 were reported as negative by all 5 technologists, 3 by 4 technologists, 1 by 3 technologists, and 5 by 1 technologist (Table 2). Of the two *Cystoisospora-*positive specimens falsely reported as negative, the majority of these incorrect results were determined from the MAF smears (MAF, *n* = 7; UV, *n* = 2). False positives were reported by the technologists for eight specimens, wherein *Cyclospora* was the coccidia described by all technologists regardless of detection method. No false positives associated with *Cystoisospora* were reported. Two of the eight false-positive specimens were reported by multiple technologists. One specimen was reported as falsely positive for *Cyclospora* by 3 technologists, technologists 1 and 3 reported it from the UV preparation only, and technologist 5 reported it from both UV and MAF slides. The second false-positive specimen associated with multiple technologists reporting *Cyclospora-*present was attributed to technologists 4 and 5. The remaining six false positives were reported by technologist 5 (3—MAF only, 2—UV only, 1—UV and MAF). Discrepant results were reported for three specimens, *Cyclospora* was misidentified as *Cystoisospora* from one specimen by technologist 4, whereas *Cystoisospora* was misidentified as *Cyclospora* for two specimens by technologists 4 and 5. All instances of misidentified coccidia were reported from the UV wet mount preparation. The presence of *Cystoisospora* in addition to *Cyclospora* was reported for a single specimen (correct result, *Cyclospora* only), by technologists 1 and 3 and only from the MAF slide.

**TABLE 1:** Detection of *Cyclospora* and *Cystoisospora* in UV wet mount and MAF slide preparations stratified by technologist experience

Specimens (*n* = 50)	Technologist					Average (95% CI)
	1	2	3	4	5	
Agreement						
UV	47 (94%)	47 (94%)	47 (94%)	44 (88%)	37 (74%)	44.4 (88.8%, 86.3%–91.3%)
MAF	43 (86%)	45 (90%)	42 (84%)	43 (86%)	40 (80%)	42.6 (85.2%, 84.2%–86.2%)
False negative						
UV	2 (4%)	3 (6%)	2 (4%)	3 (6%)	7 (14%)	3.4 (6.8%, 5.6%–8.0%)
MAF	6 (12%)	5 (10%)	6 (12%)	7 (14%)	5 (10%)	5.8 (11.6%, 11.1%–12.1%)
False positive						
UV	1 (2%)	0	1 (2%)	1 (2%)	5 (10%)	1.6 (3.2%, 2.1%–4.3%)
MAF	0	0	0	0	5 (10%)	1 (2%, 0.7%–3.3%)
Discrepant *Cystoisospora^a^*						
UV	0	0	0	1 (2%)	0	0.2 (0.4%, 0.1%–0.7%)
MAF	0	0	0	0	0	0
Discrepant *Cyclospora^b^*						
UV	0	0	0	1 (2%)	1 (2%)	0.4 (0.8%, 0.5%–1.1%)
MAF	0	0	0	0	0	0
Additional coccidian*^c^*						
MAF	1 (2%)	0	1 (2%)	0	0	0.4 (0.8%, 0.5%–1.1%)

^*a*^ *Cyclospora*-positive specimen that technologist reported *Cystoisospora* detected.

^*b*^ *Cystoisospora*-positive specimen that technologist reported *Cycylospora* detected.

^*c*^ *Cyclospora*-positive specimen that technologist reported *Cyclospora* and *Cystoisospora* detected.

**TABLE 2:** Breakdown of 13 false-negative reports of *Cyclospora* or *Cystoisospora* by method of detection stratified by technologist experience

Positive specimen (coccidian identity)	Technologist				
	1	2	3	4	5
1 (*Cyclospora*)	UV	MAF	UV	UV, MAF	MAF
2 (*Cycstoisospora*)	MAF	UV	MAF	MAF	MAF
3 (*Cyclospora*)	–^*a*^	–	–	–	UV
4 (*Cyclospora*)	UV, MAF	UV, MAF	UV, MAF	MAF	MAF
5 (*Cyclospora*)	MAF	MAF	MAF	UV	MAF
6 (*Cyclospora*)	–	–	–	–	UV
7 (*Cycstoisospora*)	MAF	–	MAF	MAF	UV
8 (*Cyclospora*)	–	–	–	–	UV
9 (*Cyclospora*)	MAF	UV, MAF	MAF	–	MAF
10 (*Cyclospora*)	–	–	–	MAF	–
11 (*Cyclospora*)	–	–	–	–	UV
12 (*Cyclospora*)	–	MAF	–	UV, MAF	UV
13 (*Cyclospora*)	MAF	–	MAF	MAF	UV

^*a*^ –, the technologist detected the organism as intended.

Eight specimens for UV wet mount and six MAF slides (total specimens, *n* = 12) associated with discordant results by the technologists were backread by a clinical parasitology expert. Results from the expert backreading were concordant for 7 out of 8 UV wet mount preparations and all MAF slides. Of the single discordant UV wet mount specimen, a false-positive *Cyclospora* result was reported by the expert for a coccidia-negative specimen, which was also reported as positive for *Cyclospora* by technologist 5.

## DISCUSSION

**Fig 1:**
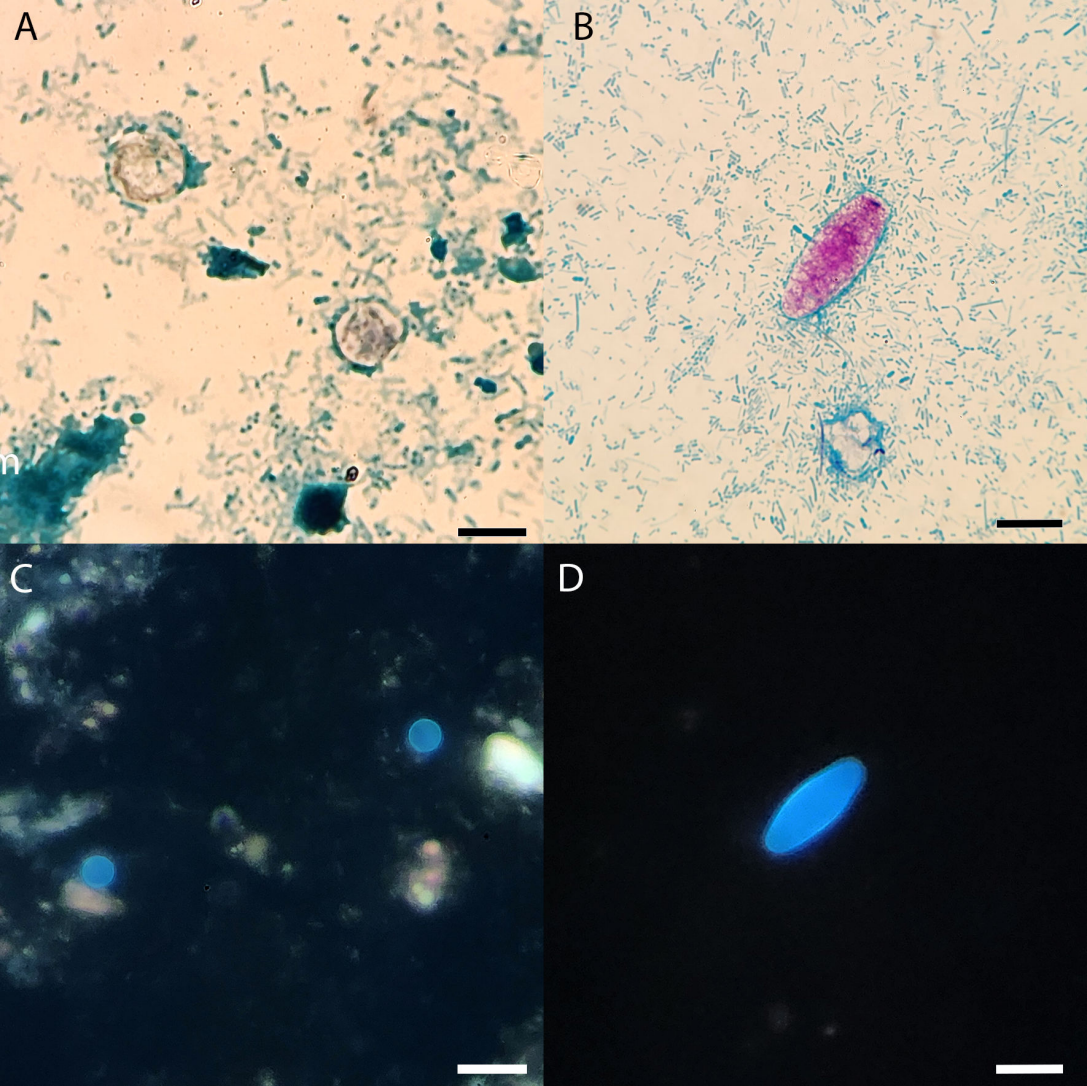
Modified acid-fast stain of *Cyclospora* (**A**) and *Cystoisospora* (**B**). UV autofluorescence using a 365 nm excitation for *Cyclospora* (**C**) and *Cystoisospora* (**D**). Scale bars indicate the following sizes: A—10 µm, B—15 µm, C—20 µm, and D—15 µm.

Stool microscopy for parasitic agents of disease remains the gold standard for the diagnosis of gastrointestinal parasites, particularly the O&P examination. Prior to the recent inclusion of *Cyclospora* in commercial multiplex NAAT assays, readily available laboratory diagnostic methods for cyclosporiasis were limited to reference laboratories that performed modified acid-fast smears or laboratory-developed NAATs. However, low-complexity laboratories may have financial or regulatory barriers to adopting commercial NAAT methods. *Cyclospora* oocysts can be occasionally detected in wet mount preparations of fresh or fixed, concentrated stool with and without iodine by light microscopy as part of routine stool O&P examinations. The oocysts range from 8 to 10 µm in size and are spherical in shape with a refractile central inclusion known as the morula (5). However, detection of *Cyclospora* in trichrome smears is challenging, due to poor uptake of stain by the oocysts, often resulting in ghost forms that make diagnosis of the parasite difficult by light microscopy without the use of the modified acid-fast or safranin stains. Modifications to traditional acid-fast stains for mycobacteria such as phenol and basic fuchsin and the inclusion of 1%–3% H_2_SO_4_ as a decolorizer instead of acid-alcohol (2) provide better dye penetration and improved visualization of the *Cyclospora* and *Cystoisospora* cell wall (Fig. 1A and B) (8). However, variable dye uptake resulting in non-uniform stained oocysts and ghost forms persist as challenges in light microscopy-based diagnosis of *Cyclospora* (Fig. 1A). Thus, the use of fluorescence microscopy to identify *Cyclospora* (and *Cystoisospora*) through their autofluorescent properties is a suitable option for diagnostic laboratories with the appropriate equipment. For *Cyclospora* and related coccidians, fluorescence microscopy without the addition of iodine (which can interfere with the autofluorescence) offers the potential benefit of increased analytical sensitivity over permanent smears. Under 365- and 450–490 nm excitation wavelengths, *Cyclospora* appears blue and green, respectively, due to tyrosine, dityrosine, and 3,4-dihydroxyphenylalanine cross-linkages in the oocyst wall (Fig. 1C) (9). Similarly, *Cystoisospora belli* and *Sarcocystis* species also possess autofluorescence properties under the same wavelengths. The improved ability for *Cyclospora* detection in fresh and fixed, concentrated stool preparations by fluorescence microscopy has been documented since 1998 (4, 10). An additional benefit, although perhaps not the intended purpose, of UV screening is the incidental detection of some helminth eggs in concentrated stool, as several nematode eggs also possess autofluorescent properties (e.g*., Trichuris, Enterobius,* hookworm, *Paracapillaria*). Although *Sarcocystis* can also be detected in UV microscopy, a lack of positive specimens rendered us unable to evaluate the performance for this pathogen.

In this study, we compare the performance of fluorescence microscopy to permanent modified acid-fast smears for *Cyclospora* detection. Overall, higher agreement and lower false-negative rates were observed for UV fluorescence microscopy than with MAF permanent smears. All technologists except for technologist 5 detected *Cyclospora* more frequently in UV preparations than in MAF slides. This may be attributed to a lower level of morphology expertise in technologist 5 who is a more recently trained medical laboratory technologist. The false positivity rate for *Cyclospora* detection was slightly higher for UV preparations, suggestive of non-specific fluorescence of cellular material or debris that may resemble *Cyclospora* in wet mount slides. There are many round, autofluorescent objects that can be seen in stool, and it is critical that careful and accurate size measurements and attention to the specific fluorescent appearance are evaluated. Only a small number of *Cystoisospora* specimens (*n* = 5) were included in this study due to the rarity of this organism in clinical practice. Relative to *Cyclospora, Cystoisospora* oocysts are larger in size (25–30 µm), and they possess a unique cylindrical shape with tapered ends that make their detection and identification by light and UV microscopy easier (Fig. 1D) (5, 11). Among the specimens that technologists reported either discrepant *Cyclospora* and *Cystoisospora* results or additional coccidia detected, it is unknown if the entity observed was a parasite or non-parasite. Of the specimens where technologists reported an incorrect, unintended coccidia, all were from UV preparations; again providing evidence of the possibility that non-parasites or autofluorescent cellular material of similar appearance was misinterpreted.

The higher instances of discrepant results associated with less experienced technologists observed in this study emphasize the importance of experience and training. This is a challenge that impacts the field of parasitology more intimately than other disciplines of clinical microbiology, as microscopy-based detection and identification of parasitic agents is not a simple binary diagnosis. Considerations must be made when analyzing specimens to determine if the entity is a parasite, non-parasite, or cellular debris and if it is indeed a parasite, whether it is pathogenic to humans that would be compatible with the clinical history and presentation (12). Clinical parasitology involves the intersection of numerous subjects including but not limited to microbiology, human physiology, and zoology. As a result, training medical laboratory technologists to be technically competent can be a laborious and time-consuming process that relies heavily on skilled mentors and continuing educational challenges through competency and clinical specimens.

This study has several strengths and limitations. In regard to strengths, we believe this to be the first detailed comparison of these two methods with multiple technologists of varying competence and experience and a well-curated, archived specimen set. In addition, this study attempts to provide data to better clarify a long-standing dogma in diagnostic parasitology which assumes increased sensitivity of UV fluorescence microscopy. Multiple commercial fixatives were also included in this study, allowing for broader applicability of the data. The acknowledged weaknesses of this study are described. First, the low incidence of cystoisosporiasis makes it difficult to evaluate larger numbers of specimens, despite our incidence being higher than contemporary reference parasitology labs (personal communications). Second, *Cyclospora,* while more prevalent than *Cystoisospora*, is still relatively uncommon in developed countries, making it challenging to collect a larger study set. Third, the nature of false-positive results cannot be easily qualified due to technologists not having imaging capabilities on their microscopes. All studies were performed in the clinical laboratory with normal patient testing environments maintained to best mimic the actual workflow and performance of these methods. As such, no photographic data exist for what was seen in false-positive results. Finally, we intentionally limited the number of negative specimens included in the study due to the intended focus on the potential increased detection of positives. By employing five different technologists, we hoped to alleviate the need to bolster the negative specimen study set while still generating additional data points.

In summary, this study provides the first (to our knowledge) direct head-to-head comparison of MAF permanent smears to UV wet mount preparations for the detection of *Cyclospora* and highlights that UV preparations are comparable and moderately superior to MAF smears. Combining UV screens with routine MAF testing on all stool specimens ordered for coccidia testing should improve overall detection rates as a result, which is critical considering the low incidence of infections encountered in many laboratories in developed countries. In our experience, performing both examinations requires minimal effort, but yields superior diagnostic yield than MAF alone. Despite advances in non-morphological testing methods in clinical parasitology, the detection of *Cyclospora* and other gastrointestinal protozoa still heavily relies on microscopic morphological detection. Microscopy offers the benefits of identifying viable infections that cannot be discerned by NAAT and a more bias-free approach toward diagnosis of diarrheal illness. In circumstances where laboratories do not have the capacity to perform permanent smears or high complexity NAATs for *Cyclospora*, those with fluorescence microscopes used for mycology or other microbiological testing should consider including UV preparations into their stool parasite testing processes routinely or when cyclosporiasis or cystoisosporiasis is clinically suspected.

## References

[1] Barratt JLN, Shen J, Houghton K, Richins T, Sapp SGH, Cama V, Arrowood MJ, Straily A, Qvarnstrom Y. 2023. Cyclospora cayetanensis comprises at least 3 species that cause human cyclosporiasis. Parasitology 150:269–285. doi:10.1017/S003118202200172X 36560856 PMC10090632

[2] Garcia LS, Arrowood M, Kokoskin E, Paltridge GP, Pillai DR, Procop GW, Ryan N, Shimizu RY, Visvesvara G. 2018. Practical guidance for clinical microbiology laboratories: laboratory diagnosis of parasites from the gastrointestinal tract. Clin Microbiol Rev 31:e00025-17. doi:10.1128/CMR.00025-17 29142079 PMC5740970

[3] Dubey JP, Khan A, Rosenthal BM. 2022. Life cycle and transmission of Cyclospora cayetanensis: knowns and unknowns. Microorganisms 10:118. doi:10.3390/microorganisms10010118 35056567 PMC8779055

[4] Mathison BA, Pritt BS. 2021. Cyclosporiasis—updates on clinical presentation, pathology, clinical diagnosis, and treatment. Microorganisms 9:1863. doi:10.3390/microorganisms9091863 34576758 PMC8471761

[5] McHardy IH, Wu M, Shimizu-Cohen R, Couturier MR, Humphries RM. 2014. Detection of intestinal protozoa in the clinical laboratory. J Clin Microbiol 52:712–720. doi:10.1128/JCM.02877-13 24197877 PMC3957779

[6] Almeria S, Chacin-Bonilla L, Maloney JG, Santin M. 2023. Cyclospora cayetanensis: a perspective (2020–2023) with emphasis on epidemiology and detection methods. Microorganisms 11:2171. doi:10.3390/microorganisms11092171 37764015 PMC10536660

[7] Kimura K, Kumar Rai S, Takemasa K, Ishibashi Y, Kawabata M, Belosevic M, Uga S. 2004. Comparison of three microscopic techniques for diagnosis of Cyclospora cayetanensis. FEMS Microbiol Lett 238:263–266. doi:10.1016/j.femsle.2004.07.045 15336431

[8] Li J, Cui Z, Qi M, Zhang L. 2020. Advances in cyclosporiasis diagnosis and therapeutic intervention. Front Cell Infect Microbiol 10:43. doi:10.3389/fcimb.2020.00043 32117814 PMC7026454

[9] Belli SI, Wallach MG, Luxford C, Davies MJ, Smith NC. 2003. Roles of tyrosine-rich precursor glycoproteins and dityrosine- and 3,4-dihydroxyphenylalanine-mediated protein cross-linking in development of the oocyst wall in the coccidian parasite Eimeria maxima. Eukaryot Cell 2:456–464. doi:10.1128/EC.2.3.456-464.2003 12796290 PMC161462

[10] Berlin OGW, Peter JB, Gagne C, Conteas CN, Ash LR. 1998. Autofluorescence and the detection of cyclospora oocysts. Emerg Infect Dis 4:127–128. doi:10.3201/eid0401.980121 9452408 PMC2627673

[11] Cama VA, Mathison BA. 2015. Infections by intestinal coccidia and Giardia duodenalis. Clin Lab Med 35:423–444. doi:10.1016/j.cll.2015.02.010 26004650 PMC4724871

[12] Bradbury RS, Sapp SGH, Potters I, Mathison BA, Frean J, Mewara A, Sheorey H, Tamarozzi F, Couturier MR, Chiodini P, Pritt B. 2022. Where have all the diagnostic morphological parasitologists gone? J Clin Microbiol 60:e0098622. doi:10.1128/jcm.00986-22 36314793 PMC9667774

